# Investing in Late-Life Brain Capital

**DOI:** 10.1093/geroni/igac016

**Published:** 2022-05-18

**Authors:** Walter D Dawson, Erin Smith, Laura Booi, Maia Mosse, Helen Lavretsky, Charles F Reynolds, Jeffrey Cummings, Patrick Brannelly, William Hynes, Eric J Lenze, Facundo Manes, Rym Ayadi, Lori Frank, Sandra Bond Chapman, Ian H Robertson, Lori Rubenstein, Jorge Jraissati, Agustin Ibáñez, Howard Fillit, Dilip V Jeste, Anitha Rao, Michael Berk, Eric A Storch, Antonella Santuccione Chadha, Harris A Eyre

**Affiliations:** Department of Neurology, School of Medicine, Oregon Health & Science University, Portland, Oregon, USA; Global Brain Health Institute at University of California, San Francisco (UCSF), San Francisco, California, USA; Trinity College Dublin, Dublin, Ireland; Global Brain Health Institute at University of California, San Francisco (UCSF), San Francisco, California, USA; Trinity College Dublin, Dublin, Ireland; Department of Medicine, Stanford Hospital, Stanford, California, USA; Global Brain Health Institute at University of California, San Francisco (UCSF), San Francisco, California, USA; Trinity College Dublin, Dublin, Ireland; Centre for Dementia Research, School of Health, Leeds Beckett University, Leeds, UK; Department of Medicine, Stanford Hospital, Stanford, California, USA; Semel Institute for Neuroscience and Human Behavior, University of California, Los Angeles (UCLA), Los Angeles, California, USA; Department of Psychiatry, University of Pittsburgh, Pittsburgh, Pennsylvania, USA; Chambers-Grundy Center for Transformative Neuroscience, Department of Brain Health, School of Integrated Health Sciences, University of Nevada, Las Vegas, Las Vegas, Nevada, USA; Alzheimer’s Disease Data Initiative, Gates Ventures, Redwood City, California, USA; Department of Medicine, Stanford Hospital, Stanford, California, USA; Department of Psychiatry, Washington University School of Medicine, St Louis, Missouri, USA; Institute of Cognitive and Translational Neuroscience (INCYT), INECO Foundation, Favaloro University, Buenos Aires, Argentina; National Scientific and Technical Research Council (CONICET), Buenos Aires, Argentina; Euro-Mediterranean Economists Association, Barcelona, Spain; RAND Corporation, Arlington, Virginia, USA; Center for BrainHealth®, The University of Texas at Dallas, Dallas, Texas, USA; Global Brain Health Institute at University of California, San Francisco (UCSF), San Francisco, California, USA; Trinity College Dublin, Dublin, Ireland; Center for BrainHealth®, The University of Texas at Dallas, Dallas, Texas, USA; Australian Research Alliance for Children and Youth (ARACY), Canberra, Australian Capital Territory, Australia; IESE Center for Public Leadership and Government, IESE Business School, Madrid, Spain; Global Brain Health Institute at University of California, San Francisco (UCSF), San Francisco, California, USA; Trinity College Dublin, Dublin, Ireland; Latin American Brain Health Institute (BrainLat), Universidad Adolfo Ibáñez, Santiago, Chile; Alzheimer’s Drug Discovery Foundation (ADDF), New York City, New York, USA; Departments of Geriatric Medicine, Palliative Care and Neuroscience, The Icahn School of Medicine at Mount Sinai, New York City, New York, USA; Departments of Psychiatry and Neurosciences, University of California San Diego, La Jolla, California, USA; IBM-UC San Diego Center for Artificial Intelligence for Healthy Living, University of California San Diego, La Jolla, California, USA; Neurocern, Chicago, Illinois, USA; Department of Neurology, University of Toledo College of Medicine and Life Sciences, Toledo, Ohio, USA; IMPACT, the Institute for Mental and Physical Health and Clinical Translation, Deakin University, Geelong, Victoria, Australia; Department of Psychiatry, University of Melbourne, Melbourne, Victoria, Australia; Department of Psychiatry and Behavioral Sciences, Baylor College of Medicine, Houston, Texas, USA; Biogen, Cambridge, Massachusetts, USA; Women’s Brain Project, Zurich, Switzerland; IMPACT, the Institute for Mental and Physical Health and Clinical Translation, Deakin University, Geelong, Victoria, Australia; Neuroscience-inspired Policy Initiative, Organisation for Economic Co-operation and Development (OECD) and the PRODEO Institute, Paris, France

**Keywords:** Aging, Brain health, Innovation, Mental disorders, Neuroscience, Psychiatry

## Abstract

Within many societies and cultures around the world, older adults are too often undervalued and underappreciated. This exacerbates many key challenges that older adults may face. It also undermines the many positive aspects of late life that are of tremendous value at both an individual and societal level. We propose a new approach to elevate health and well-being in late life by optimizing late-life Brain Capital. This form of capital prioritizes brain skills and brain health in a brain economy, which the challenges and opportunities of the 21st-century demands. This approach incorporates investing in late-life Brain Capital, developing initiatives focused on building late-life Brain Capital.

**Translational Significance:** This paper highlights and seeks to drive investments into late-life Brain Capital, which prioritizes brain skills and brain health in a brain economy. It will unlock and capitalize on the value of aging for individuals, organizations, and societies.

To capitalize on the myriad opportunities of an increasingly aging world, a new approach is needed for the 21st century (Avan et al., 2021)—one that focuses on optimizing late-life Brain Capital. Brain Capital is broadly defined as a form of capital, which prioritizes brain skills and brain health within the knowledge economy. We previously developed a Brain Capital Grand Strategy consisting of three main components: considering Brain Capital in all policies, developing a comprehensive investment plan to support Brain Capital, and the generation of a Brain Capital Index (to track and monitor Brain Capital; Eyre et al., 2021; Smith, Ali, et al., 2021). Synergistically, the Organisation for Economic Co-operation and Development’s (OECD’s) New Approaches to Economic Challenges Unit (NAEC) recently established the Neuroscience-inspired Policy Initiative, which shares a similar focus and vision (Organisation for Economic Co-operation and Development, 2021). The NAEC seeks to reconceptualize and revitalize the world economy and the way it operates. It sets forth a foundation to identify relevant metrics while concurrently building a transdisciplinary network of stakeholders who are all engaged in building the 21st-century brain economy (Council, 2014; Smith et al., 2020; Smith, Lavretsky, et al., 2021). The effort draws on experts from a wide variety of disciplines including neuroscience, medicine, gender analysis, economics, public policy, philanthropy, and business. This Initiative will catalyze, refine, and advance the concept of Brain Capital through a series of research projects, economic modeling, seminars, and policy proposals. Our Brain Capital Grand Strategy aligns with the focus of the OECD Neuroscience-inspired Policy Initiative (Smith, Ali, et al., 2021).

Here we expand on our Brain Capital Grand Strategy to include prioritizing late-life Brain Capital. Optimizing late-life Brain Capital requires a specialized focus, with policy innovations and investments in domains that support late-life health, economic security, and well-being. This paper therefore incorporates late-life Brain Capital in all policies and new investment approaches. It differs from traditional approaches in that it focuses on building late-life Brain Capital at a societal level. Thus, it seeks to facilitate retention, engagement, and empowerment of older adults, as well as compassion (e.g., support of underserved and marginalized communities), experience-derived emotional regulation (e.g., managing the adverse effects of social media on minds across the age continuum), self- reflection and self-correction, acceptance of diverse perspectives (inclusion of women, persons from racial and ethnic minorities, and older adults at leadership levels), and prosocial decision making.

This Brain Capital focus on late life encompasses individual-, corporate-, societal-, and global-level strategies for promoting and protecting brain health. Work at the level of the individual should support brain health as well as societal-level outcomes. Corporate strategies could include capitalizing on accrued wisdom and experience in mentorship and personnel development. At societal and global levels, specific opportunities to make change through policymaking—evaluating and learning from those policies—would keep the focus on effective interventions implemented in the most optimally efficient ways guided by highly experienced and “wise” older individuals. We outline here several approaches that could help facilitate the building of late-life Brain Capital.

## Investing in Late-Life Brain Capital

A range of novel investment approaches further the goal of building late-life Brain Capital and could be useful models for developing new investment approaches in the future. Table 1 focuses on investment approaches for late-life Brain Capital.

**Table 1.** Investment Approaches for the Late-Life Brain Capital Investment Plan

Approach	Process by which approach supports Brain Capital	Exemplar initiatives
Social impact investing	Outcomes-based funding strategies allow public-sector entities (e.g., governments) to pay only for what works, to the extent that it works; at the same time, they create pathways for the most impactful providers and interventions to grow if they can achieve key policy priorities. These strategies appear to be especially valuable when targeting the social determinants of health—and particularly behavioral health.	Capital Impact Partners (https://www.capitalimpact.org/) is dedicated to financing and scaling age-friendly communities nationwide, including expanding the scope and integration of the medical, social, and practical needs of older adults as they evolve. Increasingly, organizations such as Capital Impact Partners are setting a new norm for impact metrics in investing. Environmental, social, and governance factors are being weighed not only as indicators of corporate competitiveness, but also as a way to minimize long-term risk to investors’ portfolios.
Taxation and accounting restructuring to support Brain Capital	Exploring the permanent redesignation of business expenditures on employee payroll, health, reskilling, retraining, innovation, and human productive capacity as insurable capital investments. Treating expenditures on the employment, development, health, and productive capacity of people as capital investment to stimulate economic activity. This can be referred to as a Human Capital Accounting Framework, as published recently by the World Economic Forum and Willis Towers Watson (World Economic Forum, 2020).	A dichotomy exists between the idea that corporations should receive favorable tax treatment for investing in “things” but not for investing in “people” (Abbott & Bogenschneider, 2018). For example, except for some write-offs for staff costs within Research and Development work, people are typically considered a current year expense for tax purposes. In terms of cost management, that expense is often the biggest target and first to be reduced. To demonstrate the depth of this dichotomy, large workforce layoffs (people) are seen as a job loss; conversely, if the same company were to sell or close the same percentage of their physical operation (things), this would be equivalent to declaring bankruptcy or going out of business. A proposition exists to rewrite relevant tax provisions so as to make investments in people training, reskilling, health and well-being, labor contracts, and human intelligence that can facilitate the development and use of artificial intelligence, a capital expense to be written off over a period of years. Capital investments can be restructured as investments in people, thus removing the incentive for employees to be the first to go when cost-cutting measures are instituted. Workforce strategies focused on retaining aging workers in the workforce longer to offset skill and experience losses from a young-worker--centric approach. Workers themselves may wish to work longer in life for either monetary and/or purpose- driven considerations.
Government grant programs	Governmental bodies can issue grants to support Brain Capital initiatives (e.g., technological, nonprofit, etc.).	The U.S. Small Business Innovation Research (SBIR) program is a competitive program that encourages domestic small businesses to engage in Federal Research/ Research and Development with the potential for commercialization (Small Business Innovation Research, n.d.). The SBIR offers grants targeted to aging and dementia-related technologies which are available on a rolling basis similar to other federal research funding mechanisms. In June 2020, the European Commission announced a plan to increase research spending by 94.4 billion Euros over the next 7 years (Wallace, 2020). The goal of this enhanced spending is to drive productivity, employment, and competitiveness.

Table 1 has been adapted and modified from Smith, Ali et al. (2021).

The restructuring of taxation and accounting systems offers an opportunity to make investments in people through training, reskilling, health and well-being, labor contracts, human intelligence—a capital expense that businesses can write off over a period of years. Capital investments can be recast as investments in people, supporting the development of late-life Brain Capital. Taxation and accounting restructuring is especially pertinent for aging workers, as these strategies could help workforces retain older workers longer, offsetting skill and experience losses from a young-worker focus. Workers themselves may seek to work longer in late life for either monetary and/or purpose-driven considerations; taxation and accounting restructuring would help facilitate and support this “new normal.” The accrued wisdom and experience of older adults who remain in the workforce could also facilitate mentorship and personnel development within existing corporate and organizational structures. Efforts that support the continued and active participation of aging adults in the workforce are essential to building late-life Brain Capital.

Novel environment, sustainability, and governance exchange-traded funds (ESG ETFs) could provide a model for late-life Brain Capital investing. For context, an ESG ETF analyses a company not only from a traditional financial standpoint (e.g., cash flow, earnings, or debt, etc.), but also from a societal perspective, encompassing the company’s environmental, social, and political impacts. Extending ESG ETFs to human health, specifically human longevity and healthy aging, could provide a vehicle for investing in companies that promote and benefit from late-life Brain Capital.

Innovative approaches to philanthropy are another way of investing in late-life Brain Capital. The venture philanthropy model may fund breakthrough research in academia and industry that is needed to develop and protect late-life Brain Capital, such as research to prevent, slow, and treat Alzheimer’s disease and related dementias. One such example of this funding model is the Alzheimer’s Drug Discovery Foundation, which makes investments in research and treatments (Alzheimer’s Drug Discovery Foundation, 2020). Further, funds that bring together different philanthropic organizations may lead to unprecedented collaborations and investments in late-life Brain Capital. Driven by research efficacy and research outputs (e.g., patents, economic impact, and cognitive/functional outcomes) rather than monetary returns, philanthropy offers a promising model for investing in late-life Brain Capital. Return on investment of the typical venture fund becomes return on innovation in the venture philanthropy context.

Taxation and accounting restructuring, the broadening of ESG ETFs to include brain health, and novel approaches to philanthropy can lead to innovative investment approaches for late-life Brain Capital. To maximize the potential value, opportunities such as changes to taxation policy, the creation of new brain health-focused ESG ETFs, and greater collaboration from multiple global philanthropic organizations must be pursued to support late-life Brain Capital. Supplementary Table 1 provides further examples of novel investment approaches.

Several existing initiatives further the goal of building late-life Brain Capital and could provide predicates for the development of future initiatives. Table 2 focuses on initiatives for building late-life Brain Capital.

**Table 2.** Initiatives Focused on Building Late-Life Brain Capital

Initiative focused on building late-life Brain Capital	Overview of how the initiative builds late-life Brain Capital
The Davos Alzheimer’s Collaborative (DAC)	A public–private partnership working toward a global response to Alzheimer’s disease (AD) and the challenges it poses to millions of individuals and families globally. The DAC seeks to raise $700 million to fund a 6-year plan to accelerate and diversify innovation in AD-focused research. The three primary components of DAC include a global cohort developed to identify new targets for potential treatments, a global clinical trial support platform to reduce the cost and time to test new treatments in trials and bring them to market and promote health care system preparedness to get novel treatments to individuals. The DAC project will enable novel biomarker development, connect global researchers using the data platform provided by the Alzheimer’s Disease Data Initiative (ADDI), and keep people with the lived experience of AD at the center of its efforts.
Alzheimer’s Disease Data Initiative (ADDI)	ADDI, a 501(c)(3) medical research organization in partnership with the University of Washington, is dedicated to advancing scientific breakthroughs in the treatment of Alzheimer’s and related dementias (Alzheimer’s Disease Data Initiative, 2021). ADDI aims to increase interoperability of existing data platforms globally, increasethesharing of dementia-related data from academic and industry sources, and empower scientists to find, search, combine, and analyze data that could lead tonewdiscoveries in dementia research.TheADDI also aims to enhance or fill gaps in data sets, including enabling the generation of demographically representative datasets.
Alzheimer’s Drug Discovery Foundation (ADDF)	A notable model that can spur new developments in late-life brain health is that of the ADDF, which utilizes venture philanthropy to fund breakthrough research in academia and the biotech industry with promise for preventing and treating Alzheimer’s disease and other dementias (Alzheimer’s Drug Discovery Foundation, 2020). The ADDF builds partnerships across disciplines with governments, philanthropy, industry, and nonprofits including the Bill & Melinda Gates Foundation and AARP so as to advance this goal (Idrus, 2018). All proceeds that may accrue to the ADDF which result from the program’s funded research successes are in turn reinvested in new research activities.

The Global Brain Health Institute (GBHI) is an exemplar initiative of the types of innovations in education and workforce training that are needed to prepare a global community of late-life Brain Capital builders. Each year, a new global cohort of brain health leaders participate in the Atlantic Fellows for Equity in Brain Health program. Since 2015, more than 170 fellows representing 46 countries have been trained through this program and have come from a wide range of professions and disciplines across law, medicine, business, social sciences, journalism, and the arts. The program has funded 114 pilot projects, while several Atlantic Fellows have together been awarded more than $30 million to implement programs, develop new projects, and conduct research all within a shared vision of reducing the worldwide burden of dementia, which is a significant threat to the preservation and building of late-life Brain Capital.

The BrainHealth Project is an innovative example of an initiative focused on establishing preventative, population health-level interventions to protect and accelerate brain health across the life span (Chapman et al., 2021). Based on more than 30 years of research, the BrainHealth Project has developed a holistic, composite measure of brain health called the BrainHealth Index and a brain health training program that seeks to sharpen strategic attention, thinking, and lifestyle choices. The BrainHealth Project offers a proactive approach to promoting the ability to thrive in late life, protecting against mental health and cognitive decline that may either result from or occur during advanced age.

The Global Council on Brain Health (GCBH) is an independent organization, created by AARP, in collaboration with Age UK, to provide trusted information on older individuals can maintain and improve brain health. Recommendations generated by GCBH are based on the latest scientific evidence provided by scientists, doctors, scholars, and policy experts from around the world. Brain health is vital to well-being across the life span. The GCBH is organized as a hub-and-spoke collaborative to address the many factors that can affect brain health. The governance committee—comprised of individuals from around the world—is the hub. This committee identifies issue specialists—the “spokes”—to examine priority areas critical to building and maintain brain capital, including physical exercise, mental engagement, diet, supplements, sleep, stress levels, vascular risk, delirium, music, and socialization. The GCBH is an example of how organizations such as ARRP that is vitally interested in the well-being of older persons can form spin-offs and start-ups specifically devoted to Brain Capital.

The Healthy Brain Initiative of the U.S. Centers for Disease Control and Prevention (CDC) represents an example of federal governmental investment in building and preserving Brain Capital. Preserving Brain Health is presented as a central tenant of Public Health practice. The initiative creates and supports partnerships, collects and reports data, increases awareness of brain health, and develops policies to support brain health in populations with a high burden of dementia and cognitive impairment. The CDC and departments of public health have the capacity to advance brain capital by creating awareness about the interplay between brain health and physical health. Cognitive decline risk can be mitigated by public health policies addressing tobacco use, blood pressure control, cardiovascular disease management, diabetes control and prevention, obesity management and prevention, and injury prevention. These strategies can preserve Brain Capital and may help to address issues that disproportionately affect disadvantaged populations. The initiative is an example of use of government resources to advance Brain Capital.

The Women’s Brain Project (WBP) demonstrates the importance of protecting and building late-life Brain Capital among women. The WBP is a global, interdisciplinary, nonprofit organization, which has spearheaded international efforts at both the research and policy levels to understand the role of sex and gender in brain and mental health. WBP has helped to interrogate and generate evidence that sex and gender are important variables that affect the course, risk factor profile, symptom presentation, and treatment of brain disorders, as well as specific needs for care. This evidence is key to developing research and policy strategies that can be tailored to the individual, leveraging differences to promote health equality from a sex and gender perspective and paving the way for precision medicine. The WBP helps to keep women healthy as they age, benefitting society and the economy, while building late-life Brain Capital.

The GBHI, the BrainHealth Project, GCBH, CDC Healthy Brain Initiative, and the WBP all provide predicates for innovative initiatives focused on building late-life Brain Capital. Continued funding and global outreach will be key to ensuring the long-term success of initiatives like the GBHI. The BrainHealth Project will need to find ways to scale their outreach and involve people from around the world. The WBP will need to identify ways to integrate their research on the effects of sex and gender on brain health into the clinical setting. These examples are merely a subset of the challenges these initiatives will need to overcome to continue to build and optimize late-life Brain Capital.

## Conclusion

The United Nations (UN) has called this the “Decade of Healthy Ageing” (UN, 2021). The UN Resolution follows the recent endorsement of the Decade by the World Health Assembly and calls upon the World Health Organization to lead the implementation of the Decade in collaboration with other UN organizations. The UN Decade of Healthy Ageing calls for a fundamental shift in how society works, thinks, and feels about aging with multistakeholder engagement. However, the UN Decade of Healthy Ageing does not give due regard to the value of late-life Brain Capital. We align with the UN Decade of Healthy Ageing and further suggest that to unlock the value of aging and overcome key challenges associated with aging, greater prioritization of the mind and brain is needed at an individual, societal, and global level. Placing late-life Brain Capital at the center of progress and economic development will enable governments, civil societies, companies, and the international community to be better equipped with the health and skills needed to face unprecedented global challenges. Wisdom, resiliency, impact, and thriving should be part of the common cultural narratives of aging. This paper seeks to develop a strategy based on compassion, acceptance of diverse perspectives, and support for underserved populations, one that incorporates investing in late-life Brain Capital, developing initiatives focused on building late-life Brain Capital. In doing so, we hope to prioritize and optimize late-life Brain Capital and unlock and capitalize on the value of aging.

## Supplementary Material

igac016_suppl_Supplementary_Material
