# Correction to: Investing in Late-Life Brain Capital

**DOI:** 10.1093/geroni/igac042

**Published:** 2022-06-24

In the Invited Article, “Investing in Late-Life Brain Capital,” DOI: 10.1093/geroni/igac-16, co-author Patrick Brannelly’ name and was misspelled as “Brannally”. It has since been corrected.

