# Supplementary material for: Investing in Late-Life Brain Capital
Source: Innov Aging. 2022 May 18;6(3):igac016. doi: 10.1093/geroni/igac016 (PMC9116879; doi:10.1093/geroni/igac016)
Supplement: igac016_suppl_Supplementary_Material [file igac016_suppl_supplementary_material.docx]

**Supplemental Table 1: Investment Approaches for Late-Life Brain Capital Investment Plan**

| **Approach** | **Process by which Approach Supports Brain Capital** | **Exemplar Initiatives** |
| --- | --- | --- |
| Megafund | A public-private partnership to develop a portfolio approach to drug and device development in which multiple projects are undertaken simultaneously, for the purpose of developing Brain Health therapeutics. Although a greater upfront investment is required, the probability of at least one success should be higher with “multiple shots on goal,” accelerating the search for a cure and increasing the probability of one or two successes within the next decade. | Several private companies are currently using elements of the portfolio approach to the megafund for drug and device development, including therapeutics that may have Brain Health benefits. In addition, legislation toward the creation of a formal megafund has been discussed in congressional hearings. |
| Early-stage technology investment | Examples of early-stage investments include angel investors, venture capital, and private equity. These focus on investing in companies working to commercialize intellectual property for high returns. | A Venture Capital firm, Primetime Partners, launched with a focus on early-stage investments in experiences, products, and services for older adults. The firm’s interests include care management, longevity health services, financial security in retirement, and enriching consumer experiences amongst the older adult population. Additionally, Primetime Partners invests in companies founded by older adults, building on their earlier career experiences. |
| Big Data Investments | Neuroinformatics, predictive analytics, data mining, data science, computational neuroscience | Neuroinformatics is a cross disciplinary field that draws on principles from computer science, biomedicine, decision science, and information science. These methods may use a multi-modal approach to process data which can provide a more holistic brain health view. Neurocern is a neuroinformatics and predictive analytics company using large neurological and cognitive datasets that provide end users with new data and insights into brain health. For example, Neurocern’s proprietary informatics engine uses structured and unstructured data inputs (i.e., claims, prescription, or EMR data, etc.) to model risk scores for neuro-mortality, high cost of care, neuro-longevity, or eligibility for a clinical trial. Neurocern’s customizable forward facing software products use care partner as well as patient generated data along with predictive analytics to educate and help navigate populations to the right care at the right time. |
| Brain bonds | Municipal governments and sovereign issuers offer ‘social bonds’ to fund social outcomes such as research or support of clinical care (The World Bank, 2018). These types of bonds focus on economic impacts in a region or on a particular area of interest. | An example of an initiative that is developing innovative financing mechanisms in brain science which has late-life significance is the Healthy Brains Global Initiative (HBGI). This is a collaboration of global leaders from public policy, neuroscience, and finance all working together to develop a set of financing mechanisms to support brain science breakthroughs (Healthy Brains Global Initiative, 2020). Of particular interest to the HBGI is reducing the $3 trillion in global costs facing LMICs as well as HICs related to brain-based issues. While working to reduce these unsustainable costs, determining the potential feasibility of a healthy brain bond is a high priority (The World Bank, 2018; Healthy Brains Global Initiative, 2020). |
| Novel Environment, Sustainability and Governance Exchange Traded Funds | An environment, sustainability, and governance exchange-traded fund (ESG-EFT), which analyze a company’s value from a societal perspective, encompassing the company’s environmental, societal, and political impact rather than through a singularly financial-focused framework. | Several examples of specialized ESG ETFs exist in areas of health. We suggest this model could be also considered for the brain in late life. For example, investors have been developing approaches for investing in human longevity, through specialized ETFs. New funds include Global X Longevity Thematic ETF (symbol: LNGR); CI Global Longevity Economy Fund (LONG); and the Long-Term Care ETF (OLD). Axa IM, the global investment manager, also moved an existing healthcare fund into the Axa WF Framlington Longevity Economy fund. Longevity funds invest in companies that could benefit from healthy aging, financial services for longer lives, aging in place, autonomous vehicles and technologies that aid long-term services and supports or other sectors promoting healthy longevity. The core premise is a longer lifespan is only beneficial for individuals, society, and the economy if additional years of life can be enjoyed in good health and without fear of financial distress along with opportunities for continued learning, working, and leadership. |
| Special Purpose Acquisition Company (SPAC) | A special purpose acquisition company, or SPAC, is a shell company created by investors with the singular purpose of raising money via an initial public offering (IPO) to acquire another company. | Frontier Acquisition Corporation is a prime example of a SPAC focused on longevity and rejuvenation biotechnology. More SPACs with a similar focus may likely be established. |
| B Corporations | As novel from of corporate structing, a B Corporation is a business that is certified as meeting certain established standards. These standards include social and environmental performance, public transparency, and legal accountability while balancing profit and purpose.  B Corporations also seek to shift global culture that redefines what is accepted as business success, while building an economy that is far more inclusive and sustainable. | The B Corporation community supports reducing inequality and poverty, while also supporting a healthier environment, stronger communications, and the creation of more high-quality jobs for workers that provide dignity and purpose. B Corporations seek to leverage their profits and growth as a means to a greater end such as positive impacts for their employees, communities, and the environment. Examples of B Corporations within the aging field include Renewal Memory Partners and Home Care Associates of Philadelphia. A searchable list of all certified B Corporations can be found on their website directory (<https://bcorporation.net/directory>). |
| Philanthropy | Philanthropy may have objectives for specific diseases, institutions, or populations that could benefit from research. Driven by research efficacy and research outputs (e.g., patents, economic impact, and patient outcomes, not monetary returns). | The CARE Fund (<https://carefund.org/>) reflects public demand for changes pertaining to care, with eight major foundations including the Ford Foundation, Robert Wood Johnson Foundation and W.K. Kellogg Foundation pooling an initial $50 million USD investment to support a broad-based movement for care. This includes paid leave, childcare and early education, long-term services and support for older adults and people living with disabilities, and high-quality jobs for all care workers. This collaborative fund will focus its investments in broad-based movement-building to amplify the voices of those receiving and delivering care; advocating for policy and change; implementing new policies and investments at the community level to support children and families; winning dignified living wages and benefits; and redefining both care work and care services as a permanent public good worthy of financing and investing in. |

Supplementary Table 1 has been adapted and modified from Smith, Ali et al. (2021).
